# Quality of life and satisfaction of patients after oncoplastic or traditional breast-conserving surgery using the BREAST-Q (BCT module): a prospective study

**DOI:** 10.1007/s12282-023-01474-1

**Published:** 2023-06-26

**Authors:** M. Ghilli, M. D. Mariniello, F. Ferrè, R. Morganti, E. Perre, R. Novaro, L. Colizzi, V. Camilleri, G. Baldetti, E. Rossetti, L. Coletti, C. Scatena, M. Ghilardi, M. C. Cossu, M. Roncella

**Affiliations:** 1 Breast Centre AOUP, University Hospital of Pisa, Pisa, Italy; 2 Scuola Superiore Sant’Anna of Pisa, Laboratorio Management E Sanità, Istituto di Management, Pisa, Italy; 3 Unit of Statistics, University Hospital of Pisa, Pisa, Italy

**Keywords:** BREAST-Q, Breast cancer, Breast-conserving therapy, Oncoplastic breast surgery, Patient-reported outcome measures

## Abstract

**Introduction:**

The oncoplastic conservative surgery was developed as a natural evolution of traditional surgery, attempting to improve the therapeutic and aesthetic outcomes where tumor resection could be followed by not-adequate results. Our primary aim is to evaluate how patient satisfaction and quality-of-life after conservative oncoplastic surgery, using BREAST-Q (BCT Module), change pre- and post-operatively. The secondary aim is to compare patient-reported outcome after oncoplastic or traditional conservative surgery.

**Patients and methods:**

We enrolled 647 patients who underwent traditional conservative surgery or oncoplastic surgery from January 2020 to December 2022. Only 232 women (35.9%) completed the BREAST-Q questionnaire on a web-based platform, at the preoperative phase and 3 months after treatment.

**Results:**

The average score of “Psychosocial well-being” and “Satisfaction with Breasts” 3 months after surgery showed a statistically significant improvement, while the average score for “Physical well-being: Chest” at 3 months showed a worsening compared to the baseline. “Sexual well-being” did not show statistically significant change. A significant difference between the post-operative outcome of oncoplastic surgery and traditional surgery was observed only for Physical well-being (better for traditional surgery).

**Conclusions:**

The study showed significant improvement in patient-reported outcomes 3 months after the surgery, except for physical discomfort that increases especially after oncoplastic surgery. Furthermore, our data, as well as many others, point to the appropriateness of using OCS where there is an effective indication, while the perspective of patients cannot find significant superiority over TCS in any of the areas analyzed.

## Introduction

Oncoplastic conservative surgery (OCS) has progressively developed as the natural evolution of traditional breast-conserving surgery (TCS) or quadrantectomy, extending the role of surgery where tumor resection was not practicable (except with a mastectomy) or followed by inadequate cosmetic outcomes, to obtain better aesthetic and surgical results with negative resection margins [1]. The modern definition of oncoplastic surgery is “a form of breast conservation surgery that includes oncologic resection with a partial mastectomy, ipsilateral reconstruction using volume displacement or volume replacement techniques with possible contralateral symmetry surgery when appropriate” [2]. Surgical outcomes were confirmed by the low long-term relapse rate, comparable to mastectomy [3-5] OCS, embodying both terms of oncological resection and plastic reconstruction, guarantees a reduced incidence of local recurrences, achieving a more accurate tumor resection than simple quadrantectomy [6], more satisfactory cosmetic results, and less need for a second surgery [7].

Moreover, there is strong evidence about the better acceptability of OCS versus the mastectomies and reconstruction: OCS appears superior to mastectomy in terms of satisfaction with breasts, sexual well-being, and psychosocial well-being [8].

Research in breast surgery has significantly evolved in the last decade, since the medical community has tried to go beyond the assessment of traditional outcome, such as morbidity and mortality. On this track, patient-reported outcome measures (PROMs), in which the patient's perception of the results is quantified, have become increasingly important, as the breast surgery aims to improve satisfaction, psychosocial well-being, and quality of life (QoL). One of the most used is the BREAST-Q, an international tool developed and validated by the Memorial Sloan Kettering Cancer Center (New York, USA), for the evaluation of patients’ satisfaction following surgical treatment [9-11]. As main aspects, it investigates QoL (psychosocial, physical, and sexual well-being) and satisfaction (with breasts, outcome, and care). Since its development in 2009, BREAST-Q has provided meaningful and reliable information on health, QoL (including physical, psychosocial, and sexual well-being) and patient satisfaction, thus proving usefulness in both clinical and research practice. It also made it possible to measure, from the patient's perspective, the quality revealed by surgical staff in different types of surgery [12]. The BREAST-Q Breast-Conserving-Therapy (BCT) module proved to be a reliable questionnaire for the assessment of QoL and patient satisfaction after conservative surgery in breast cancer (BC) patients [9, 13, 14]. This questionnaire can also be used as a tool to help health-professionals and patients make a decision about which breast surgery should be adopted to improve patients' satisfaction with their physical appearance, consistently with their desires and expectations [15].

The BREAST-Q-(BCT) has already successfully administered in some international studies showing how much patients undergoing OCS were satisfied with their breasts (also compared to controlateral breast in unilateral surgeries) [16].

The systematic use of BREAST-Q is still limited, but few years ago, the Breast Unit of Pisa (Italy) started the continuous, systematic, and digital collection of PROMs, accordingly with the quality requirements [17]. The Breast Unit is collecting BREAST-Q score for patients undergoing breast reconstruction after mastectomy as part of a regionally based observatory of patients’ outcome and experience coordinated by the Management and Healthcare Laboratory (MeS Lab) of the Scuola Superiore Sant’Anna, Pisa [18-20]. More recently, the Breast Unit has also started the collection of BREAST-Q-BCT module.

## Patients and methods

### Study aim

The primary aim of this study is to evaluate the QoL and the satisfaction of the patients who underwent TCS or OCS through the online administration of the BREAST-Q Breast-Conserving-Therapy (BCT) Module at the preoperative phase (t0) and 3 months after surgery (t1) [14]. It is also planned a t2 evaluation 12 months after surgery (still ongoing). As a second aim, the study evaluates any difference between TCS and OCS, through a comparison of the scores of the BREAST-Q considering that the two approaches were used in different groups of patients. Indeed, the indications to TCS or OCS mainly are based on the site and the size of the disease and also the type of breast (ptosis and size) and the patients’ preferences (i.e., rejection of a proposed symmetrization or request of a reduction mammoplasty even when not necessary for a correct tumor excision).

In this study, differences in outcome for women undergoing any adjuvant chemotherapy and radiotherapy are not considered due to the short follow-up. Patients submitted to neoadjuvant chemotherapy are not included in the study.

### Study setting and survey

This is a prospective nonrandomized single-center study on two cohorts of BC patients who underwent, respectively, TCS or OCS from January 2020 to December 2022 at the University Hospital of Pisa. The Breast Unit accounts for about 730 interventions for new diagnosed BC each year, of whom about 65% are conservative. The study obtained the approval of the local Ethics Committee (ONCBR-27/02/2020). The data used in this paper include preoperative and post-operative satisfaction and QoL scores collected with the BREAST-Q. The post-operative scores refer to outcome at t3 moths after surgery (t1). Informed consent was obtained from all participants.

The BREAST-Q is a disease-specific validated questionnaire for assessing PROMs [10], evaluating the psychosocial, physical, and sexual well-being, and satisfaction with breasts [21]. The questionnaire was administered online using the information system developed by the MeS Lab-Sant'Anna of Pisa for the regionally based Observatory on patients’ outcome and experience. Once the patients filled in the questionnaire, the scores are automatically computed and reported in an aggregate form on a platform accessible by professionals and healthcare managers. A BREAST-Q conversion table transforms raw data into summary scores ranging from 0 (very dissatisfied) to 100 (very satisfied).

### Patients

647 patients treated for invasive primary BC from January 2020 to December 2022 were recruited: 346 underwent TCS and 301 OCS. The length of hospital stay after surgery was < 24 h for almost all the TCS and < 48 h for almost all OCS, and accordingly, the frequency of post-operative analgesic use was slightly greater for OCS.

### Statistical analysis

Categorical data were described by absolute and relative frequency, continuous data by mean and standard deviation. To analyze continuous variables between preoperative (t0) and post-operative (t1), we used *t* test for paired data (two-tailed). Furthermore, ANOVA for repeated measures stratified for surgery type was performed. Significance was fixed at 0.05. All statistical analyses were carried out by SPSS v.27 technology.

## Results

### Patients, comorbidities, and treatment factors

Response rate was 35.9%: although almost all patients completed at least one questionnaire, only 232 of the 647 enrolled patients completed both the questionnaires at the preoperative and post-operative phase. A t2 questionnaire is being completed by patients 1 year after surgery and will be analyzed in a further paper.

Mean age at surgery was 58 years (sd 11 years). Within this sample, 31% (*n* = 172) had at least one comorbidity excluding BC, and the remaining did not report any comorbidity.

143 patients underwent monolateral surgery, 89 bilateral surgery (80 due to controlateral symmetrization and 9 for bilateral cancer). 128 patients underwent traditional quadrantectomy (TCS), 104 oncoplastic surgery (21 monolateral, 83 bilateral). Sentinel lymph-node biopsy was performed in 87.5% (*n* = 203), axillary dissection ab initio in 9.9% (*n* = 23), and both procedures (more than two sentinel lymph nodes positive for metastasis) in 1.3% (*n* = 3).

Patients’ comorbidities and treatment approaches are summarized in Table 1.

**Table 1:** Clinical and surgical characteristics of the population. Statistics: frequency (%) or mean (SD)

Characteristics	All women (232 patients)	Women with TCS (128 patients)	Women with OCS (104 patients)
Age	58 (11)	59 (12)	57 (11)
*Performance status*			
No comorbidities	160 (69%)	82 (64%)	78 (75%)
At least one comorbidity	72 (31%)	46 (36%)	26 (25%)
*Breasts involved in surgery*			
Monolateral surgery	143 (61.6%)	125 (97.7%)	18 (17.3%)
Bilateral surgery for bilateral breast cancer	9 (3.9%)	3 (2.3%)	6 (5.8%)
Bilateral surgery for controlateral symmetrization	80 (34.5%)	0 (0%)	80 (76.9%)
*Type of surgery*			
Standard quadrantectomy (TCS)	128 (55.2%)	128 (100%)	
Monolateral oncoplastic surgery (OCS)	21 (9.1%)		21 (20.2%)
Bilateral oncoplastic surgery (OCS)	83 (35.7%)		83 (79.8%)
*Axillary treatment*			
Axillary biopsy alone	203 (87.5%)	116 (90.6%)	87 (83.7%)
Axillary dissection alone	23 (9.9%)	10 (7.8%)	13 (12.5%)
Biopsy + dissection	3 (1.3%)	1 (0.8%)	2 (1.9%)
Missing	3 (1.3%)	1 (0.8%)	2 (1.9%)

*TCS* traditional breast-conserving surgery, *OCS* oncoplastic conservative surgery

### Psychosocial well-being

**Fig. 1:**
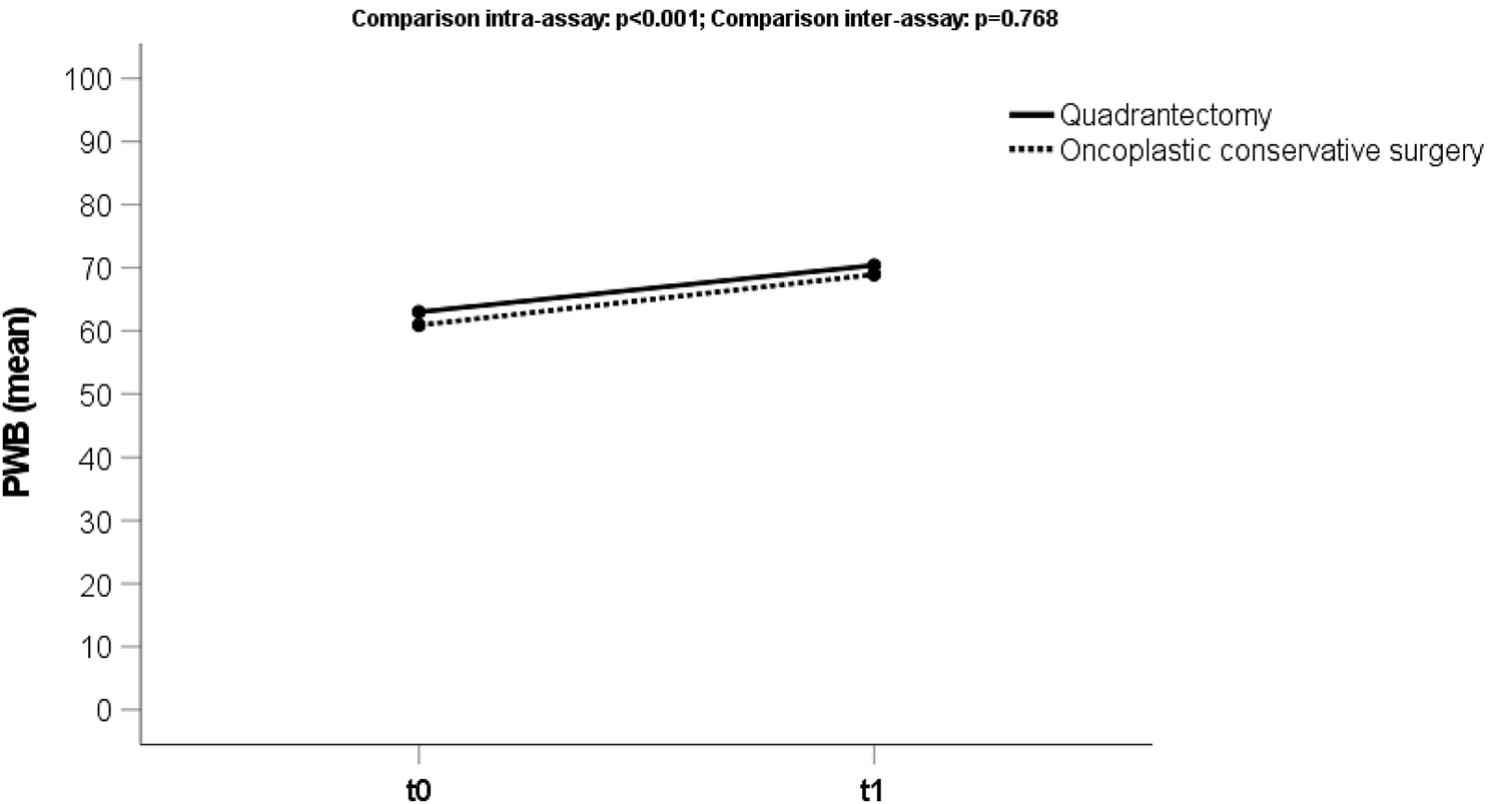
Trend score of the domain “Psychosocial Well-Being” (PWB) analyzing the preoperative phase (t0) and 3 months after treatment (t1), both for oncoplastic and traditional conserving surgery (quadrantectomy)

The average score for the domain “Psychosocial well-being” (Fig. 1) was 62.1 (sd 14) at the preoperative phase and 69.7 (sd 17) at t1. We observed a statistically significant change (*p* < 0.001) for both groups of patients (OCS and TCS) comparing t0 and t1.

Moreover, no statistically significant difference in outcome for the OCS, compared to TCS cohort (*p* = 0.298), was found.

### Physical well-being

**Fig. 2:**
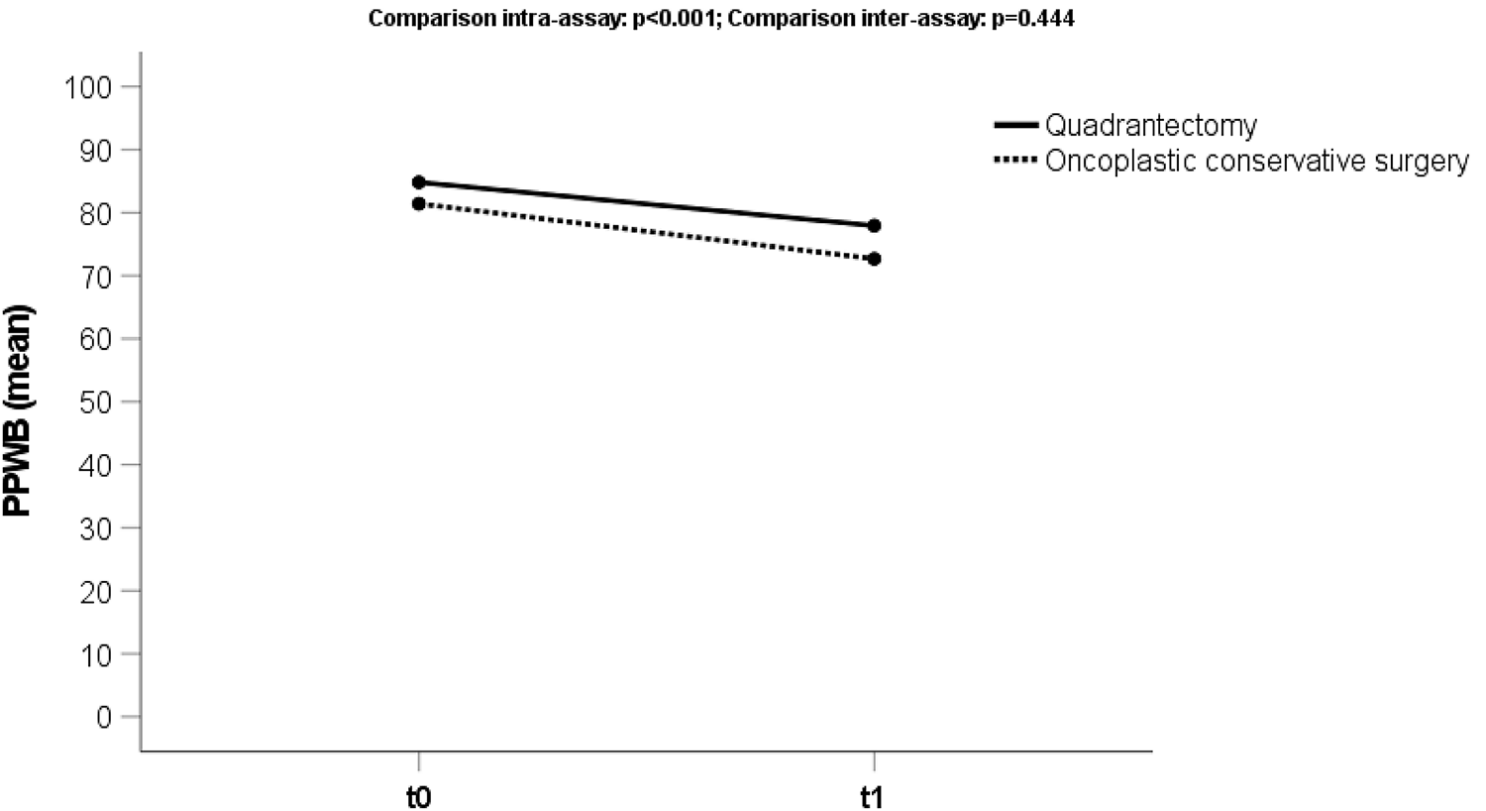
Trend score of the domain “Physical Well-Being: Chest” (PPWB) analyzing the preoperative phase (t0) and 3 months after treatment (t1), both for oncoplastic and traditional conserving surgery (quadrantectomy)

The average score for “Physical well-being: chest” (Fig. 2) was 83.2 (sd 16) preoperatively and 75.5 (sd 18) at t1. We observed a statistically significant worsening (*p* < 0.001) for both groups (OCS and TCS) comparing the preoperative and post-operative scores.

Moreover, there was a statistically significant better outcome for the TCS cohort (78.5) than OCS cohort (71.7) at the post-operative phase (*p* = 0.005).

### Satisfaction with breasts

**Fig. 3:**
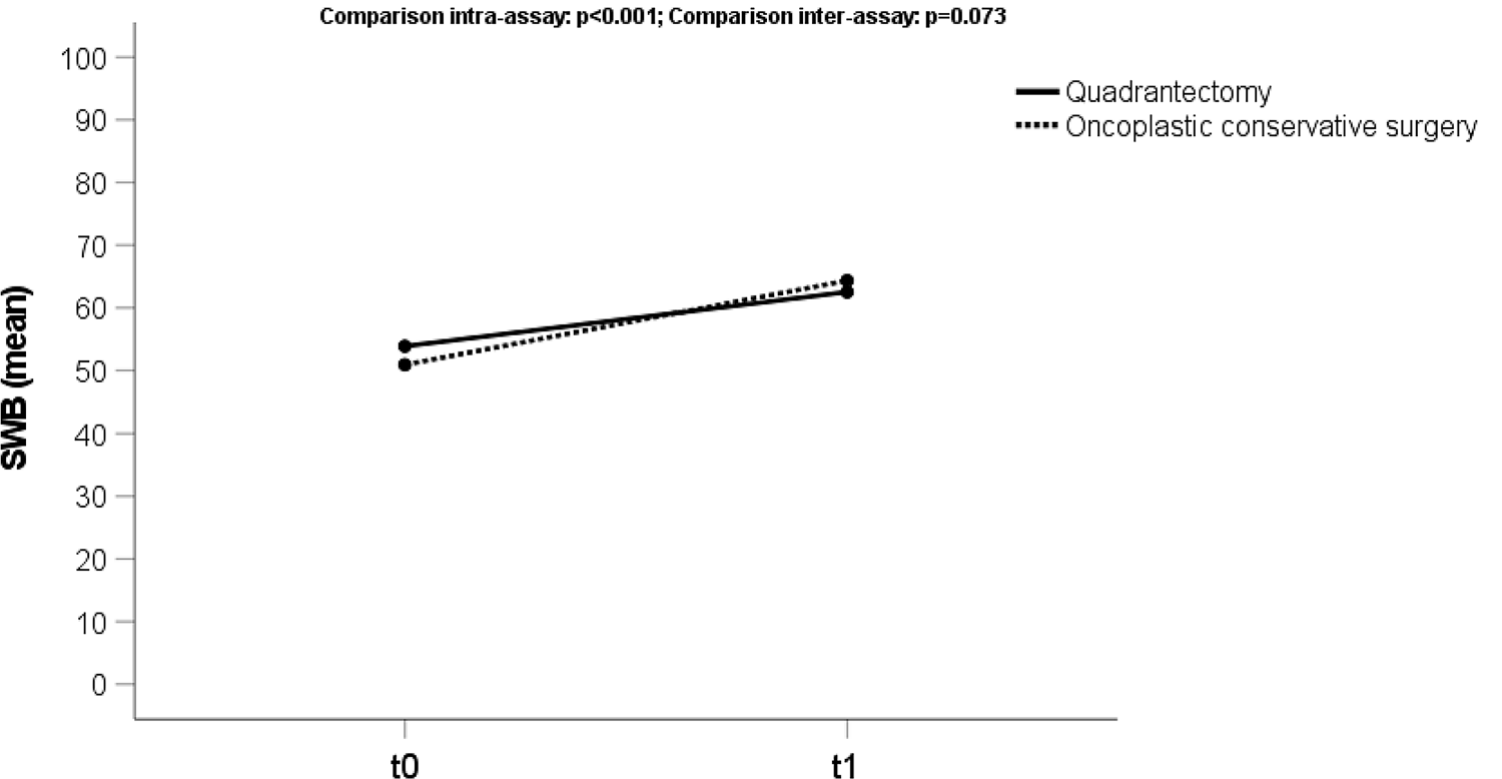
Trend score of the domain “Satisfaction with breasts” (SWB) analyzing the preoperative phase (t0) and 3 months after treatment (t1), both for oncoplastic and traditional conserving surgery (quadrantectomy)

The average score for “Satisfaction with Breasts” (Fig. 3) was 52.3 (sd 13) at t0 and 63.3 (sd 16) at t1. We observed a statistically significant change (*p* < 0.001) for both groups of patients (OCS and TCS) comparing the preoperative and post-operative questionnaires’ scores.

Moreover, there was a better outcome (64.1) for the OCS cohort than TCS (62.7) at t1, even though the difference is not statistically significant (*p* = 0.523).

### Sexual well-being

**Fig. 4:**
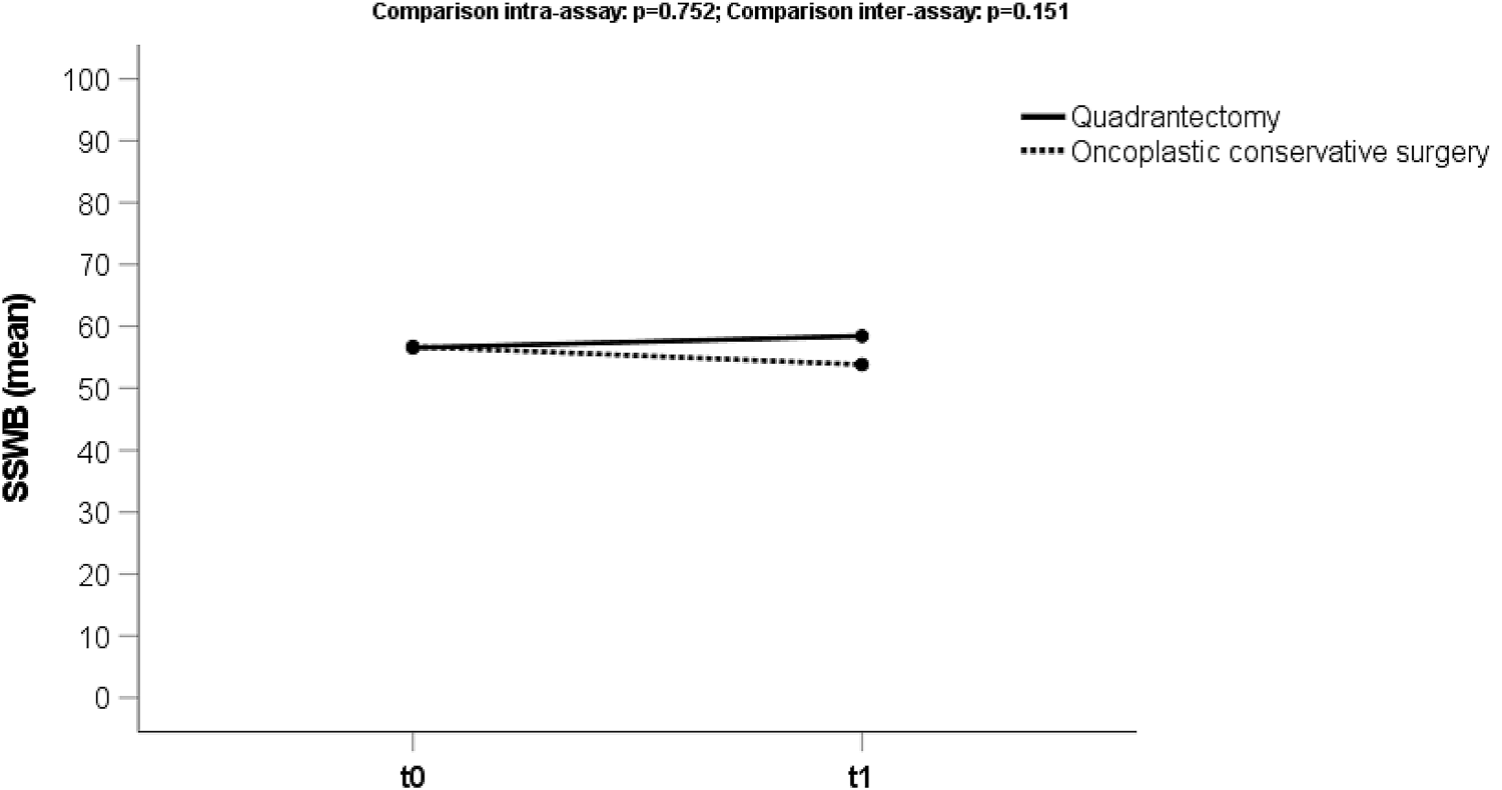
Trend score of the domain “Sexual Well-Being” (SSWB) analyzing the preoperative phase (t0) and 3 months after treatment (t1), both for oncoplastic and traditional conserving surgery (quadrantectomy)

The median score for the domain “Sexual well-being” (Fig. 4) was 56.7 (sd 17) at t0 and 56.0 (sd 22) at t1. We observed a worst outcome in the OCS (53.0) than the TCS group (58.6), although it was not statistically significant (*p* value 0.100).

## Discussion

Oncoplastic surgery has been developed to overcome aesthetical limitations that TCS had shown for patients with certain oncological situations (e.g., big tumor size that requires an extensive excision of the breast) [22-24] and anatomical patterns (breast size, tumor/breast ratio, and tumor site, especially in inner and lower quadrants) [5, 25-28]. The OCS has demonstrated a high patient satisfaction as cosmetic outcome [29, 30], while it ensures an oncological efficacy, confirmed by the low recurrence rates evidenced with a longer follow-up so far [3, 4, 7, 27]. The BREAST-Q proved to be one of the most suitable tools to assess in a standardized way, through pre- and post-operative scales, the QoL, and the satisfaction of patients following breast surgery [10]. The first experiences were in post-mastectomy reconstruction and more recently also in conservative surgery [13-16, 29, 31].

We evaluated the outcomes of OCS or TCS using BREAST-Q BCT module. The response rate in this trial was less than 40%: although almost all patients completed at least one questionnaire, only 232 of 647 enrolled filled in both the questionnaires t0 and t1. In previous experiences, the response rate resulted higher, and this is probably due to the more recent introduction of the BCS scale (with respect to post-mastectomy reconstructive module) in the clinical pathway in our center, and in any case, it appears acceptable. A study reported 83% of response rate but only on 144 patients and only assessed post-operatively and retrospectively [16]; another is based on a large Danish registry with response rate of at least 48% again administered only post-operatively [29]. Finally, a review describes 38 studies reporting a response rate ranging from 32 to 100%, but it includes almost only post-mastectomy patients, using a more broad definition of oncoplasty (including also mastectomy, reconstruction, and additional symmetrisation procedures either immediately after the procedure or at a later date). Providing the design of our trial also a 1-year (t2) questionnaire (that will be analyzed at the end of 2023), some patients responded/will respond to that but not to t1 or t0. Finally, the pandemic has significantly impacted the study, with the consequent difficulty, especially in the first months, of enrolling patients.

Some aesthetic and functional outcomes of OCS and TCS have been investigated before [32] and there are only a few studies evaluating QoL related to TCS [10, 31, 33], OCS [16], or both [29]. The BREAST-Q BCT module was introduced in 2015 and, 1 year later, O’Connel published the initial experience with the BCT post-operative module in a retrospective study including 200 patients who had undergone unilateral BCT in the previous years [31]. In 2019, Gardjfell evaluated post-operatively the satisfaction outcome of 144 patients who underwent unilateral oncoplastic volume displacement surgery for BC, with the BREAST-Q BCT module [16].

In our study, average score for “Psychosocial well-being” at t1 is 70, significantly lower than the scores of 82 and 87 reported in O’Connel and Gardjfell. Likewise, the average score of the “Satisfaction with Breasts” was 63.3, lower than that obtained in the previous studies (68 and 77 in O’Connel and Gardfjell, respectively). The main differences between these three experiences can be referred to the design of studies: O’Connel, like Gardjfell, evaluated the outcomes through the BREAST-Q BCT post-operative module alone, implementing retrospective studies and administering the questionnaire to a cohort of patients with the surgery performed even many years before (between 2010 and 2016) [16, 31]. Compared to these, a prospective study is more reliable, as it is less affected by the many variables that may have modified, over the years, the outcome of the results (mainly recall bias).

Considering the average score of “Physical Well Being: Chest”, described a t1, our study found 75.5, in line with the scores reported by O’Connel (75) and Gardfjell (78). Conservative surgery (both OCS and TCS) have a negative impact on “Physical Well-Being: Chest”: the difference between t0 and t1 showed a decreasing value associated with an increased discomfort in the operated area (e.g., tightness, pulling, tenderness, and pain) and discomfort. It is not only a problem of conservative surgery but of breast surgery in general, obviously. Even more so, we found in our previous paper focused on BREAST-Q after post-mastectomy reconstruction that all the analyzed domains worsened post-operatively, and the differences were statistically significant for physical and sexual well-being [19].

Indeed, comparable to the aforementioned studies, a low score was highlighted in the domain “Sexual Well-Being” (56) at t1. Any decrease in sexuality score may be due not only to psychological blues after the diagnosis of cancer, but also to those factors whose prevalence increases in patients as the onset of menopause, psychological causes, and sexual dysfunction of various kinds included iatrogenic ones.

OCS appears superior to mastectomy in terms of satisfaction with breasts, sexual well-being, and psychosocial well-being [8]. Although the differences in terms of functional and aesthetic outcomes have been previously investigated through retrospective studies, there is a lack of trials comparing QoL outcomes after TCS or OCS prospectively [29, 32]. For this reason, as secondary aim of this trial, we evaluated PROMs’ difference between these groups, through the BREAST-Q (BCT).

Although the sample size led to some limitations, we evaluated in this study the different outcome between OCS and TCS in the various domains proposed. We observed a significant increase in physical discomfort among OCS compared to TCS [in “Physical well-being: chest” a statistically significant better outcome for the TCS (78.5) than OCS (71.7) at t1 (*p* = 0.005) was found], and thus, the worsening is greater for the OCS, especially in level II-techniques (defined as the necessary excision of 20–50% of the breast), which require an extensive reshaping to obtain a correct glandular mobilization, in moderate–large breasts with moderate–severe ptosis. For eligible BC patients, OCS has proven to be an optimal option to ensure satisfactory aesthetic outcome, while maintaining adequate margins [5]. By removing large volumes of tissue without a simultaneous decrease in cosmetic outcomes, OCS was indeed introduced as a standard of care in patients suitable for conservative surgery as an alternative to bad quadrantectomy or useless and excessive mastectomy. Involved procedures, however, are more complex, time-consuming, and expensive: these techniques present issues concerning safety, local control, delayed start of adjuvant treatments due to increased rate of complications, and cost-effectiveness [34]. For sure, TCS remains the best choice especially but not exclusively in case of little cancer in outer quadrants, in medium-sized breast, and in this sense, OCS and TCS should not be seen as alternatives at all but different treatments available for different groups of patients.

Moreover, in this trial, the mean score for “Psychosocial well-being” showed a higher post-operative value for TCS vs OCS, even though the difference among the two groups was not statistically significant (*p* = 0.298). Again, the minor invasiveness of the surgical approach can explain the result.

In conclusion, it has been recently stated that OCS requires accurate evaluation with validated tools to provide valuable insight into the effectiveness from both clinical and patient-reported experience and that objective measurement of the complex interaction of psychological and surgical-specific issues affecting the QoL is limited [35]. Thus, to improve outcomes in this area, the existing tools should be used to enhance data accuracy and reliability in terms of QoL and aesthetic outcomes. Moreover, the reviewed literature suggests that using author-generated questionnaires, often not validated to measure outcomes, makes it difficult to compare studies. The strength of our prospective trial can rely so far to the systematic use of validated PROMs in the under-evaluated setting of conservative surgery. Moreover, we have made a comparison between OCS and TCS, showing that the latter still has important spaces of employment. The weakness could be represented by the sample size of the patients that completed both (pre-op and post-op) questionnaires, which in any case appears among the most considerable in the available literature.

This study demonstrates that also BREAST-Q-BCT module is useful to evaluate patients’ perception in terms of sexual, emotional, and psychosocial well-being following different surgical techniques. Furthermore, our data, as well as many others, point to the appropriateness of using OCS where there is an effective indication, while it is more complex and even the perspective of patients cannot find significant superiority over TCS in any of the areas analyzed [35].

## Data Availability

Data are available for consultation and can be requested to the corrisponding author at m.ghilli@ao-pisa.toscana.it
